# The anticancer immune response of anti-PD-1/PD-L1 and the genetic determinants of response to anti-PD-1/PD-L1 antibodies in cancer patients

**DOI:** 10.18632/oncotarget.5107

**Published:** 2015-08-05

**Authors:** Xinbing Sui, Junhong Ma, Weidong Han, Xian Wang, Yong Fang, Da Li, Hongming Pan, Li Zhang

**Affiliations:** ^1^ Department of Medical Oncology, Sir Run Run Shaw Hospital, Zhejiang University, Hangzhou, China; ^2^ Biomedical Research Center and Key Laboratory of Biotherapy of Zhejiang Province, Hangzhou, China; ^3^ Department of Immunology, University of Toronto, Toronto, ON, Canada; ^4^ Transplantation Institute and Toronto General Research Institute, University Health Network, Toronto, Canada; ^5^ Department of Gastrointestinal Surgery, Nankai Hospital, Nankai District, Tianjin, China

**Keywords:** PD-1, PD-L1, cancer, checkpoint inhibitor, immunotherapy

## Abstract

The programmed death-1 (PD-1), a coinhibitory receptor expressed on activated T cells and B cells, is demonstrated to induce an immune-mediated response and play a critical role in tumor initiation and development. The cancer patients harboring PD-1 or PD ligand 1 (PD-L1) protein expression have often a poor prognosis and clinical outcome. Currently, targeting PD-1 pathway as a potential new anticancer strategy is attracting more and more attention in cancer treatment. Several monoclonal antibodies against PD-1 or PD-L1 have been reported to enhance anticancer immune responses and induce tumor cell death. Nonetheless, the precise molecular mechanisms by which PD-1 affects various cancers remain elusive. Moreover, this therapy is not effective for all the cancer patients and only a fraction of patients respond to the antibodies targeting PD-1 or PD-L1, indicating these antibodies may only works in a subset of certain cancers. Thus, understanding the novel function of PD-1 and genetic determinants of response to anti-PD-1 therapy will allow us to develop a more effective and individualized immunotherapeutic strategy for cancer.

Based on the concept that cancer cells may employ several mechanisms to escape immune recognition and elimination of their host, cancer immunotherapy is developed [1]. The aim of cancer immunotherapy is to activate a patient's own immune system to kill the tumor cells [2]. Over the past several decades, a large number of immunotherapeutic approaches to cancer treatment have been established, including cancer vaccines, adoptive transfer of ex vivo activated T and natural killer cells, anticancer monoclonal antibodies and the checkpoint inhibitors such as anti-PD-1 [3].

PD-1 (also CD279), a coinhibitory receptor expressed on the surface of activated T cells and B cells, has been linked to immune tolerance and therefore provides a possible mechanism of escape immune surveillance when tumor cells become capable of expressing PD-L1 [4, 5]. PD-1 is mainly activated by interacting with its ligands PD-L1 and PD-L2 [6, 7]. PD-L1 is widely distributed on diverse cell types in lymphoid and nonlymphoid tissues, whereas PD-L2 is mainly expressed on dendritic cells (DCs) and some macrophages [8, 9]. Once activated, PD-1 exerts a negative effect on immune responses by dephosphorylating key downstream proteins of the antigen receptor [10, 11]. Thus, the PD-1 pathway may serve as an important regulator for the induction and maintenance of peripheral immune tolerance.

PD-1 expression by tumor-infiltrating lymphocytes (TILs) was proved to correlate with impaired immune responses and poor outcome in several tumor types [12, 13]. However, there is a controversial report regarding the prognostic implication of PD-1 positive (+) TILs in cancer patients. Kim and colleagues found that increased numbers of PD-1(+) TILs were significantly associated with prolonged disease-free survival of pulmonary squamous cell carcinoma (SqCC) patients [14]. So the role of PD-1(+) TILs in the prognosis of cancer patients remains unresolved. The expression of PD-L1 has been shown to be correlated with poor prognosis in patients with non-small-cell lung cancer (NSCLC), breast cancer, gastric cancer, soft tissue sarcomas and meningioma [15-19]. Tumor cells may upregulate PD-L1 expression as a way to suppress the host immune response and therefore escape immune destruction. These data provide a clearer understanding of the PD-1/PD-L1 pathway that limits an antitumor immune response and lead to the development of several anticancer drugs by blocking the distinct checkpoints PD-1 or its major ligand PD-L1. As a potential new anticancer strategy, the checkpoint inhibitors anti-PD-1 and anti-PD-L1 have attracted an enormous amount of interest and generated an encouraging clinical outcome in the treatment of patients with solid tumors, particularly in NSCLC, renal cell carcinoma and melanoma [20, 21]. However, not all the cancer patients can gain the benefit from this treatment. For instance, anti-PD-1 antibody produced objective responses only in approximately one in four to one in five patients with NSCLC, melanoma, or renal cell cancer [22], indicating that the genetic background of cancer patients might determine the clinical responses to anti-PD-1 and anti-PD-L1 treatments. The answer to this critical issue will provide us increased understanding of response to anti-PD-1 therapy and promote the individualized application of these agents in the clinic.

In this review we will summarize the antineoplastic properties of PD-1 pathway as a rheostat of immunological regulation and discuss the genetic determinants of manipulating this strategy for cancer therapy.

## THE ROLE OF PD-1/PD-L1 PATHWAY IN CELL IMMUNE RESPONSE

**Figure 1:**
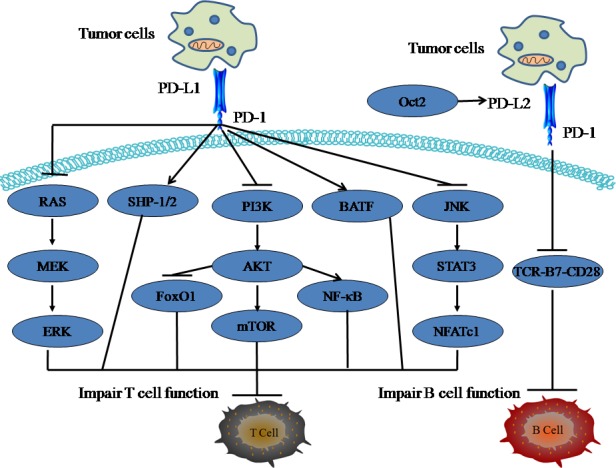
The role of PD-1/PD-L1 pathway in cell immune response PD-1 functions to inhibit T cell activation not only by attenuating TCR signaling (SHP-1/2), but also by enhancing the expression of genes that impair T cell function. PI3K-Akt-mTOR, JNK, and Ras-MEK-ERK signals are crucial regulators for PD-1-mediated inhibitory effect on T cell immune. PD-L2 is mainly expressed on B cells. Oct2 can regulate PD-L2 gene expression in B-1 cells and at low antigen concentrations, PD-L2-PD-1interactions suppressed B cell function by inhibiting TCR-B7-CD28 signals.

PD-1, a member of the immunoglobulin (Ig) superfamily, contains an immunoreceptor tyrosine-based inhibitory motif (ITIM) as well as an immunoreceptor tyrosine switch motif (ITSM) in its cytoplasmic tail and delivers inhibitory signals to immune cells upon the binding of its ligand, PD-L1 or PD-L2 [23]. PD-1 is expressed on peripheral T cells, B cells, natural killer T (NKT) cells, DCs and some monocytes upon their activation [24]. The immediate outcome of PD-1 engagement via binding with its ligand is inhibition of cell growth and cytokine secretion (Figure 1).

PD-1 engagement may directly inhibit T cell antigen receptor (TCR)-mediated effector functions within tissues via recruiting phosphatases such as SHP-1 and SHP-2 [25], which is essential for the maintenance of immune tolerance. PD-1 negatively regulates innate immune cells, the failure of which can cause the activation of autoimmunity [26]. Interactions between PD-1 and PD-L1 promoted tolerance by blocking the TCR-induced stop signal in the pancreatic islets, and blockade of PD-1 or PD-L1 suppressed T cell motility and abrogated peripheral tolerance [27]. *In vivo*, PD-1 deficiency induced autoimmunity in variety of spontaneous autoimmune diseases depending on the genetic background of the mice. The mice genetically lacking PD-1 developed dilated cardiomyopathy through the exhibition of high-titer circulating IgG autoantibodies to troponin I on the BALB/c background [28, 29]. PD-1 deficiency specifically accelerated the onset and frequency of type I diabetes in nonobese diabetic (NOD) mice [30]. Overexpression of PD-1 in DCs inhibited allogeneic lymphocyte activation in BALB/c mice [31]. Those observations suggest that PD-1 may regulate both innate responses and lymphocyte responses to prevent autoimmunity.

PD-1 functions to limit T cell activation not only by attenuating TCR signaling, but also by enhancing the expression of genes that impair T cell function. PD-1 coordinately upregulated the expression of transcription factor ATF-like (BATF) which was sufficient to impair T cell proliferation and cytokine secretion [32]. Silencing BATF in T cells reduced PD-1 inhibition and rescued HIV-specific T cell function. PD-1 altered T-cell metabolic reprogramming by inhibiting glycolysis and promoting lipolysis and fatty acid oxidation, which reveal a metabolic mechanism responsible for PD-1-mediated blockade of T-cell effector function [33]. Triggering of PD-1 inhibited T cell expansion and function by upregulating IL-10 production [34]. Blockade of PD-1 increased the activation of phosphatidylinositol-3-kinase (PI3K) and its downstream targets AKT and mammalian target of rapamycin (mTOR), which are impaired in antiviral cytotoxic T lymphocytes (CTLs). In the further study, the transcription factor FoxO1 was demonstrated to be responsible for sustaining the expression and function of PD-1 in exhausted CTLs [35]. Some other transcription factors are also involved in regulation of PD-1 expression. Mutation of Nuclear factor of activated T cells c1 (NFATc1) resulted in a complete loss of PD-1 expression in T cells [36]. The interferon (IFN)-sensitive responsive element (ISRE) was shown to be essential for IFN-alpha-induced upregulation of PD-1 in macrophages [37]. NF-κB regulated PD-1 expression in macrophages through binding the site located upstream of the gene in conserved region C [38]. In addition, PD-1 can be selectively triggered by ligation of Toll-like receptor 9, which contributes to peripheral tolerance and autoimmunity [39]. PD-1 also affected cell cycle progression and proliferation of T lymphocytes by suppressing the ubiquitin ligase SCF (Skp2) through inhibiting PI3K-Akt and Ras-mitogen-activated and extracellular signal-regulated kinase kinase (MEK)-extracellular signal-regulated kinase (ERK) signaling pathways [40].

PD-L1 is induced on various cell types in response to certain inflammatory cytokines (primarily IFN-γ), which are produced during the immune responses of T and natural killer (NK) cells [41]. IFN-γ-stimulated neutrophils suppressed lymphocyte proliferation through expression of PD-L1 [42]. The common gamma-chain cytokines IL-2, IL-7, IL-15, and IL-21 induced the expression of PD-L1, resulting in suppressing certain effector functions of cytokine-stimulated cells upon TCR engagement [43]. The inflammatory cytokines (such as IL-17 and IFN-γ)-triggered up-regulation of PD-L1 on RPE played a critical factor for inducing infiltrated uveitogenic T cells with regulatory activities [44]. Recent studies indicate that the Janus kinase (JAK)/STAT and mitogen-activated protein kinase (MAPK) signaling pathways were involved in IFN-induced PD-L1 expression [45, 46]. Signal transducer and activator of transcription 3 (STAT3) with binding to the CD274 gene promoter was also required for PD-L1 gene expression [47], moreover, STAT3-dependent upregulation of PD-L1 mediated immune regulatory functions of liver plasmacytoid DCs [48].

PD-L2, a second ligand for PD-1, is mainly expressed on B cells, DCs, macrophages, and cultured bone marrow (BM)-derived mast cells. Engagement of PD-1 by PD-L2 may dramatically suppress TCR-mediated proliferation and cytokine production by CD4+ T cells. The expression of PD-L2 also varies depending on the antigen concentrations [7]. At low antigen concentrations, PD-L2-PD-1interactions suppressed TCR-B7-CD28 signals. In contrast, at high antigen concentrations, PD-L2-PD-1 interactions attenuated cytokine production but did not inhibit T cell proliferation. There are few reports about transcriptional regulation of PD-L2. Octamer binding protein 2 (Oct2) was demonstrated to regulate PD-L2 gene expression in B-1 cells through lineage-specific activity of a unique, intronic promoter [49]. Subsequently, PD-L2 cross-linking induced NF-κB -dependent protection of dendritic cells from cell death [50].

## THE ROLE OF PD-1/PD-L1 PATHWAY IN CANCER

The PD-1/PD-L1 pathway has a crucial role in regulating immunosurveillance for tumors. PD-1 can interfere with TCR/CD28 signals to suppress the immune responses of T-cell help (Tc1/Th1 skewing) in the tumor microenvironment through the PD-1/SHP-2/p-STAT1/T-bet axis [51]. Tumor cells expressing PD-1 can limit the activity of tumor antigens (TA)-specific CD8^+^ T cells, which reinforces their growth and invasiveness [52]. PD-1 is upregulated by dysfunctional TA-specific CD8^+^ T cells both *in vitro* and *in vivo* [53], and PD-1 blockade enhances TA-specific T cell responses and inhibits tumor growth or partial tumor regression [54]. PD-1 blockade also increases T-cell migration to tumors by elevating IFN-γ inducible chemokines, which augments T-cell-mediated antitumor responses [55]. In addition, the majority of TILs predominantly express high levels of PD-1 and are thought to be correlated with an “exhausted” phenotype and impaired antitumor immune responses [56]. This “exhausted” phenotype is marked by decreased T cell proliferation, poor cytolytic activity, and low production of type I cytokines.

PD-L1 and PD-L2 expression are up-regulated in a variety of human cancer types. PD-L1 is frequently expressed in several types of solid tumor cells, whereas PD-L2 is highly expressed in certain subsets of B cell lymphomas [57-59]. Expression of PD-L1 protein significantly correlates with the levels of elevated TILs, which is associated with cancer metastasis [60]. Transgenic expression of PD-L1 in immunogenic tumor cells confers them a potent escaping from host T cell immunity and markedly enhances their invasiveness in vivo [61]. PD-L1 is also upregulated in tumors by activation of key signaling pathways including PI3K, STAT3, IFN-γ and so on. Latent membrane protein 1 (LMP1) and IFN-γ upregulate PD-L1 through STAT3, AP-1, and NF-κB pathways, which promotes progression of nasopharyngeal carcinoma (NPC) and ovarian cancer [41, 62]. The activation of MAPK promotes PD-L1 expression that is transcriptionally modulated by c-Jun and augmented by STAT3 [63]. Similarly, PD-L2 expression is observed in a subset of tumor types but its role in cancer is far less prevalent than PD-L1. PD-L2 expression in pulmonary squamous cell carcinoma is associated with an increased number of CD8^+^ TILs and proto-oncogene MET protein overexpression [14].

## PD-1/PD-L1 BLOCKADE AND ITS CLINICAL APPLICATION

Based on the concept that the blockade of PD-1 or its ligands has immune-potentiating effects on cancer cells, many monoclonal antibodies targeting PD-1/PD-L1 pathway have been developed for the treatment of various cancer types (Table 1). Among these anti-PD-1 antibodies, nivolumab and pembrolizumab, have been approved by the US Food and Drug Administration (FDA) for the treatment of patients with metastatic melanoma.

**Table 1:** Currently used anti-PD-1 and anti-PD-L1 antibodies

Target	Name	Molecule	Manufacturer	Phase
anti-PD-1	Nivolumab	Fully human IgG4	Bristol-Myers Squibb	I-III
	Pembrolizumab	Humanized IgG4	Merck& Dohme	I-III
	Pidilizumab	Humanized IgG1	CureTech	I-II
anti-PD-L1	MPDL3280A	Engineered human IgG1	Roche/Genentech	I-III
	BMS-936559	Humanized IgG4	Bristol-Myers Squibb	I
	MEDI4736	Engineered human IgG1	MedImmune	I-III
	MSB0010718C	Fully human IgG1	EMD Serono	I-II

### Nivolumab

Nivolumab (also known as ONO-4538, BMS-936558 or MDX-1106) is a genetically engineered, fully human immunoglobulin (Ig) G_4_ immune checkpoint inhibitor specifically targeting for human PD-1. The antibody binds to PD-1 with high affinity, thereby attenuating inhibitory signals and enhancing the host antitumor immune responses. Nivolumab has anticancer potential in a variety of tumor types, including melanoma, NSCLC, prostate cancer, renal cell carcinoma (RCC), Hodgkin's lymphoma and colorectal cancer (CRC). The first report of the safety and antitumor activity of nivolumab was a phase I dose escalation trial [64]. In this study, nivolumab was showed to have significant antitumor activity but a maximum tolerated dose was not confirmed. Since then, a large amount of clinic trials investigate the association between nivolumab exposure and various cancer types. Recently, Nivolumab was approved by the US FDA in December 2014 for the treatment of patients with unresectable or metastatic melanoma. Nivolumab was also approved by the US FDA for the treatment of patients with metastatic squamous NSCLC that returns during or after treatment with platinum-based chemotherapy in March 2015.

Melanoma is considered an “immunogenic” tumor and high levels of PD-L1 are frequently expressed in melanomas, leading to activation of PD-1 and downregulation of anticancer immunity [65]. Thus, immune checkpoint antibodies may have antitumor potential for patients with melanoma (Table 2). A randomized phase I clinical trial showed that nivolumab treatment could significantly improve the median progression-free survival (PFS), overall survival (OS) and objective response rate (ORR) [66]. Importantly, the responses were durable and long-term safety was acceptable. To date, no authoritative phase II clinical trials had been reported. A phase III study evaluated nivolumab versus dacarbazine in previously untreated melanoma without BRAF mutation [67]. This study suggested nivolumab significantly improved the OS and PFS in previously untreated patients who had metastatic melanoma without a BRAF mutation. Another randomised, controlled, open-label, phase III trial assessed the efficacy and safety of nivolumab versus investigator's choice of chemotherapy (ICC) as a second-line or later-line treatment in patients with advanced melanoma [68]. As a result, ORR in nivolumab patients was reported to be higher than that in ICC patients. Moreover, BRAF wild-type patients treated with nivolumab showed a better ORR than that in BRAF^V600^ mutation-positive patients. This clinical trial indicated that nivolumab represented a new treatment option for the patients with advanced melanoma that has progressed after ipilimumab or ipilimumab and a BRAF inhibitor.

**Table 2:** Efficacy data of anti-PD-1 and anti-PD-L1 antibodies

Antibody	Phase	Patients (Number)	Dose	Tumor	ORR%	Median OS, months (95% CI)	PFS months (95% CI)	Reference
Nivolumab	I	107	0.1-10mg/kg	Advanced melanoma	31	16.8 (12.5-31.6)	3.7 (1.9-9.1)	66
	III	418	3 mg/kg	Advanced melanoma	40	Unknown	5.1 (0.34-0.56)	67
	III	631	3 mg/kg	Advanced melanoma	31.7	Unknown	4.7 (2.3-6.5)	68
	I	129	1,3,10 mg/kg	Advanced NSCLC	17	9.9 (7.8-12.4)	2.3 (1.8-3.7)	70
	II	117	3 mg/kg	Squamous NSCLC	14.5	8.2 (6.1-10.9)	1.9 (1.8-3.2)	71
	I	34	1 or 10 mg/kg	Advanced RCC	29	22.4 (12.5-NE)	7.3 (3.6-10.9)	73
	II	168	0.3, 2, 10 mg/kg	Advanced RCC	20 for 0.3 22 for 2 20 for 10	20 for 0.3 22 for 2 20 for 10	18.2 for 0.3 25.5 for 2 24.7for 10	74
	I	23	3 mg/kg	Hodgkin's lymphoma	87	Unknown	Unknown	58
Pembrolizumab	I	173	2 or 10 mg/kg	Advanced melanoma	26	Unknown	5.5 for 2 3.5 for 10	75
	III	834	10 mg/kg	Advanced melanoma	33.7 for every 2 weeks 32.9 every 3 weeks	Unknown	Unknown	76
	I	495	2 or 10 mg/kg	Advanced NSCLC	19.4	12	3.7	77
Pidilizumab	I	17	0.2 to 6.0 mg/kg	Hematolog-ical malignancies	Unknown	Unknown	Unknown	79
	II	66	1.5 mg/kg	DLBCL	51	Unknown	Unknown	80
	II	32	3 mg/kg	Lymphoma	66	Unknown	Unknown	81

Similar to melanoma, NSCLC displays high expression of PD-1 or PD-L1 [69] and blocking PD-1/PD-L1 pathway as a therapeutical strategy was recently evaluated in the patients with NSCLC (Table 2). The phase I dose-escalation cohort expansion trial showed nivolumab is effective and safe for the patients with previously treated advanced NSCLC [70]. A phase II, single-arm trial indicated a positive activity of nivolumab for the patients with advanced, refractory, squamous NSCLC [71]. These data support that nivolumab has antitumor activity and a manageable safety profile in previously treated patients with advanced, refractory, squamous NSCLC. On the basis of these encouraging results, phase III clinical trials are further evaluating the activity of nivolumab in patients with NSCLC.

RCC is considered as an immunogenic tumor with dysfunctional immune cell infiltrate and PD-L1 expression correlates with increased risk of disease progression and cancer-specific death of the patients with this cancer type [72]. Thus, PD-1 can function as an emerging therapeutic target in RCC (Table 2). A phase I study with expansion cohorts evaluated clinical activity, survival, and long-term safety in patients with advanced RCC treated with nivolumab [73]. As a result, median OS (22.4 months) is encouraging, and toxicities were generally manageable. The randomized phase II trial assessed the antitumor activity, dose-response relationship, and safety of nivolumab in patients with metastatic RCC [74]. This report demonstrated nivolumab had antitumor activity with a manageable safety profile across the three doses studied in metastatic RCC. A number of ongoing phase III studies that will further elucidate this evidence.

In addition, nivolumab emerges antitumor potential in Hodgkin's lymphoma (Table 2). To determine whether nivolumab could inhibit tumor immune evasion in patients with Hodgkin's lymphoma, 23 patients with relapsed or refractory Hodgkin's lymphoma received the treatment with nivolumab. As a result, nivolumab had substantial therapeutic activity and an acceptable safety profile in patients with previously heavily treated relapsed or refractory Hodgkin's lymphoma [58]. Several results from phase II and III clinical trials are awaited yet.

### Pembrolizumab

Pembrolizumab (MK-3475), a humanized IgG_4_ monoclonal antibody blocking the interaction of PD-1 on T cells with its ligands, is believed to reactivate antitumor immunity (Table 2). A phase I trial assessed the efficacy and safety of pembrolizumab in patients with ipilimumab-refractory advanced melanoma [75]. This study suggested that pembrolizumab could be an effective treatment option for patients with ipilimumab-refractory advanced melanoma. The randomized, controlled, phase III trial compared pembrolizumab with ipilimumab in 834 patients with advanced melanoma [76]. The results showed that pembrolizumab could prolong PFS and OS and had less high-grade toxicity than did ipilimumab in patients with advanced melanoma.

In NSCLC, the efficacy and safety of pembrolizumab is currently being investigated in a phase I trial [77]. It was indicated that pembrolizumab had antitumor activity in patients with advanced NSCLC, moreover, PD-L1 expression in at least 50% of tumor cells was shown to be correlated with improved efficacy of pembrolizumab. In addition, pembrolizumab is currently being investigated in a phase I/II trial in the patients with some other cancer types, including breast cancer, bladder cancer, and haematologic malignancies.

### Pidilizumab

Pidilizumab (CT-011) is a humanized IgG_1_ kappa recombinant monoclonal antibody against PD-1. In preclinical studies, pidilizumab was demonstrated to inhibit cancer cells survival (Table 2) [78]. In a phase I clinical trial, pidilizumab was shown to be safe and well tolerated in patients with advanced hematological malignancies, and no single maximum tolerated dose was defined in this study [79]. Two phase II trials assessed the safety and activity. One evaluated the antitumor activity of pidilizumab in diffuse large B-cell lymphoma (DLBCL) [80]. The other trial enrolled 32 patients with relapsed follicular lymphoma and these patients were received the treatment with the combination of pidilizumab and rituximab [81]. These two trials suggested pidilizumab was worthy of further study in follicular lymphoma. Currently, the efficacy and safety of pidilizumab is underway in other tumor types.

### Other anti-PD-L1 antibodies

MPDL3280A, an engineered human anti-PD-L1 monoclonal IgG_1_ antibody, has noteworthy antitumor activity in metastatic urothelial bladder cancer (UBC) [82]. MPDL3280A is currently being investigated in combination with dabrafenib, vemurafenib or trametinib in advanced melanoma with or without BRAF mutation. The phase II/III trials of MPDL3280A in advanced NSCLC, metastatic RCC, and breast cancer are also ongoing.

MEDI4736, an engineered fully-human anti-PD-L1 antibody, has been reported to have an ORR in 13% across all the patients with lung cancer, but up to 39% in PD-L1^+^ patients and 5% in PD-L1 negative patients [83]. Based on these outcomes in lung cancer, several clinical trials for MEDI4736, both as monotherapy as well as in combination with other agents, is underway across a range of tumor types.

## THE GENETIC DETERMINANTS OF ANTI-PD-1/PD-L1 ANTIBODIES RESPONSE

Although a number of studies have reported that anti-PD-1/PD-L1 antibodies exhibit antitumor immune responses in variety of cancer types, significant interindividual variability in response attenuates its optimal use. This may attribute to the various genetic backgrounds of the different tumor types. Thus, it will be important to understand why some cancer patients are sensitive to these antibodies, what are the genetic determinants of response to this therapy.

Although early studies indicated that PD-L1 expression may be a predictable biomarker for therapeutic response to anti-PD-1 antibodies, only a subset of patients with NSCLC responded to PD-1 blockade, indicating the genetic background of the cancer patients may be a key determinant of response to this therapy. PD-L1 expression in at least 50% of NSCLC cells correlated with improved efficacy of pembrolizumab [77]. Recently, it was shown that high PD-L1 expression was associated with the presence of epidermal growth factor receptor (EGFR) mutation in advanced lung adenocarcinoma and PD-L1 overexpression was considered as a poor prognostic indicator in EGFR wild-type patients but not in EGFR mutant patients [84]. Another study demonstrated that EGFR mutant tumors display elevated PD-L1 levels and the EGFR mutant mouse showed significant response to the treatment with anti-PD-1 antibody, indicating EGFR mutation may be a promising biomarker of response to this treatment [85]. In addition, higher nonsynonymous mutation burden, the molecular smoking signature, higher neoantigen burden, and DNA repair pathway mutations were also showed to determine sensitivity to PD-1 blockade in NSCLC [86]. Moreover, long-term clinical benefit from anti-PD-L1 therapy in lung cancer was also discovered to be associated with JAK3 activation [87].

PD-L1 expression in advanced melanoma was thought to be correlated with response to monoclonal antibodies targeting PD-1/PD-L1 pathway, moreover, the sensitivity to PD-1/PD-L1 blocking antibodies seemed to be more robust in patients whose tumors express PD-L1 [88, 89]. However, the potential relationship between the BRAF mutation and PD-L1 expression has not been determined. Several recent reports demonstrated that PD-L1 expression did not correlate with BRAF mutational status in melanoma and lung adenocarcinomas [90, 91]. Thus, the BRAF mutational status does not play a major role in directly determining the efficiency of PD-1 pathway blockade in melanoma cells. Recently, NRAS mutations in advanced melanoma were correlated with increased benefit from anti-PD-1/PD-L1 therapy compared with other genetic subtypes [92].

PD-L1 mRNA expression was identified to be associated with increased tumor-infiltrating immune cells and better outcome in breast carcinomas [93]. In patients with RCC, the presence of PD-1(+) tumor-infiltrating immune cells was associated with more aggressive tumors and shortened survival [12]. The responses of MPDL3280A were observed in patients with high expression of PD-L1, especially when PD-L1 was expressed by tumor-infiltrating immune cells. In addition, T-helper type 1 (T_H_1) gene expression, CTLA4 expression and the absence of fractalkine (CX3CL1) in baseline tumor specimens were also related with the response to the anti-PD-L1 antibody MPDL3280A in cancer patients [94]. The pre-existing CD8+ T cells were shown to be associated with expression of the PD-1/PD-L1 immune inhibitory axis and may predict response to PD-1 blockade [95]. The mismatch repair deficiency is also considered as a genomic marker to predict response to PD-1 blockade with pembrolizumab in colorectal and other cancers [96].

Taken together, these molecular targets are important for design of future clinical trials assessing the antitumor potential of anti-PD-1/PD-L1 antibodies.

## CONCLUSION AND THERAPEUTIC PERSPECTIVES

Immune checkpoint modulation as a therapeutic strategy is attracting more and more attention in cancer therapy. Increasing data from pre-clinical studies and clinical trials have confirmed that anti-PD-1/PD-L1 antibodies have antitumor potential and improve cancer patients' survival. Currently, anti-PD-1 or anti-PD-L1 therapy is under individual study and in combination with other therapies such as cytotoxic chemotherapy, antiangiogenic agents and small-molecule tyrosine kinase inhibitors.

Nevertheless, this treatment raises many issues. First, it is not exactly clear how PD-1 mediates their effects since the possible molecular mechanisms by which anti-PD-1/PD-L1 antibodies enhance the host antitumor immune responses remain elusive. Secondly, to maximize the potential to be applied for more stringent clinical study, the efficacy and safety of PD-1/PD-L1 targeting antibodies should be further investigated. Thirdly, the determinants of the immune responses of these agents are still unknown. Thus, it is urgent to discern more sensitive and specific predictors of clinical outcomes in order to identify patients who will benefit the most from the clinical treatment. However, our increased understanding of PD-1 mediated signal pathways will hopefully broaden the number of therapeutic targets and perhaps provide a prospective strategy for cancer by modulating the immune checkpoints.
